# Anti-Obesity Effects of *Sargassum thunbergii* via Downregulation of Adipogenesis Gene and Upregulation of Thermogenic Genes in High-Fat Diet-Induced Obese Mice

**DOI:** 10.3390/nu12113325

**Published:** 2020-10-29

**Authors:** Min-Cheol Kang, Hyo-Geun Lee, Hyun-Soo Kim, Kyung-Mo Song, Yong-Gi Chun, Min Hyeock Lee, Bum-Keun Kim, You-Jin Jeon

**Affiliations:** 1 Research Group of Process Engineering, Korea Food Research Institute, Jeollabuk-do 55365, Korea; mckang@kfri.re.kr (M.-C.K.); mhlee@kfri.re.kr (K.-M.S.); hyogeunlee92@gmail.com (Y.-G.C.); gustn783@mabik.re.kr (M.H.L.); 2 Department of Marine Life Science, Jeju National University, Jeju 63243, Korea; rudah@kfri.re.kr; 3 Department of Genetic Resources Research, National Marine Biodiversity Institute of Korea, Janghang-eup 33662, Korea; ygchun@kfri.re.kr

**Keywords:** anti-obesity, *Sargassum thunbergia*, seaweed, high-fat diet

## Abstract

Obesity is a metabolic disease characterized by an increased risk of type 2 diabetes, hypertension, and cardiovascular disease. We have previously reported that compounds isolated from brown alga, *Sargassum thunbergii* (ST; *Sargassum thunbergii* (Mertens ex Roth) Kuntze), inhibit adipogenesis in 3T3-L1 cells. However, the in vivo anti-obesity effects of these compounds have not been previously reported. Therefore, the objective of this study was to determine the effects of ST on weight loss, fat accumulation, as well as risk factors for type 2 diabetes and cardiovascular disease in high-fat diet (HFD)-induced obese mice. ST treatment significantly decreased body weight and fat accumulation in HFD-induced obese mice, while reducing insulin and factors related to cardiovascular diseases (triglyceride and total cholesterol) in serum. ST-induced downregulation of PPARγ in white adipose tissue, and upregulation of the thermogenic genes, *UCP-1* and *UCP-3*, in brown adipose tissue was also observed. In addition, oral administration of ST reduced the occurrence of fatty liver, as well as the amount of white adipose tissue in HFD mice. Cumulatively, these results suggest that ST exerts anti-obesity effects and may serve as a potential anti-obesity therapeutic agent.

## 1. Introduction

Globally, the number of obese individuals increased by more than three-fold between 1980 and 2014 with more than 600 million people considered to be obese [1,2]. Obesity is characterized by an excessive accumulation of body fat and is considered a risk factor for chronic diseases and metabolic syndromes, including hypertension, type 2 diabetes, arthritis, and cardiovascular disease [3]. Currently used synthetic anti-obesity drugs, including orlistat and sibutramine, elicit certain adverse effects, including insomnia, dry mouth, dizziness, palpitations, hand tremors and elevated blood pressure [4]. A need, therefore, exists for the development of novel, safe, and effective anti-obesity agents. To this end, many studies have focused on identifying natural substances capable of eliciting anti-obesity effects. In fact, various natural compounds have recently been reported to induce anti-obesity effects via inhibition of fat accumulation and adipogenesis [5,6,7]. 

Compound derivatives from natural substances have demonstrated significant protective effects against metabolic syndromes, including diabetes, hypertension, and obesity [8,9,10]. Specifically, seaweed contains various secondary metabolites with important biological activities, including anti-oxidant, anti-cancer, anti-inflammatory, and anti-diabetic properties [11,12,13]. Similarly, naturally-derived polyphenols have demonstrated anti-oxidant, anti-inflammatory, anti-cancer, anti-bacterial, and hepato-protective effects. Moreover, previous studies have reported that polyphenol intake improves metabolic syndromes, including type 2 diabetes, hypertension, obesity, and dyslipidemia [14,15]. *Sargassum thunbergii* (ST), which is an edible brown algae, is abundant within the subtidal regions of Korea, Japan, and China; it also reportedly has anti-oxidant, anti-allergy, and anti-inflammatory properties [16,17,18]. We previously reported that compound derivatives isolated from ST inhibit adipogenesis in adipocytes, demonstrating that these compounds elicit potential anti-obesity effects in adipocytes [19]. However, no in vivo studies have yet examined the anti-obesity properties of ST. Herein, we, therefore, investigated the anti-obesity activities of ST extract in a high-fat diet (HFD)-induced obese mouse model. 

## 2. Materials and Methods

### 2.1. Preparation of Ethanol Extract from Sargassum thunbergii

ST was collected from the coast of Jeju Island, South Korea. Salt, sand, and epiphytes were removed using tap water. The freeze-dried seaweed samples were ground and passed through a 40–50 mesh by Pin-mill. Following this, 100 g of powdered ST was subjected to extraction with 70% ethanol solution at room temperature for 24 h. The extracts were subsequently concentrated and freeze-dried to obtain a 70% ethanoic extract of ST. The resulting powder was used for all subsequent studies.

### 2.2. Animals

Male C57BL/6 mice (aged 6 weeks, weighing 19–22 g) were purchased from Jung Ang Lab Animal Inc. (Seoul, Korea). Prior to use, the animals were acclimated to their environment at 22 °C with 55% humidity and a 12-h light/dark cycle for one week. The chow diet consisted of nitrogen-free extract (60.7%), proteins (15.2%), cellulose (4.1%), mineral ash (5.0%), moisture (12.1%), and lipids (2.9%) with a caloric intake of 2793 kcal. The HFD comprised casein, 30 mesh (800 kcal), L-cystein (12 kcal), corn starch (291 kcal), maltodextrin 10 (400 kcal), sucrose (691 kcal), soybean oil (225 kcal), lard (1598 kcal), vitamin mix S10026 (0 kcal), dicalcium phosphate (0 kcal), calcium carbonate (0 kcal), potassium citrate, 1 H_2_O (0 kcal), vitamin mix V10001 (0 kcal), choline bitarrate (0 kcal), federal food, drug, and dcosmetic act (FD&C) Red Dye #40 (0 kcal), with a total caloric intake of 4507 kcal. The mice were randomly divided into six groups (five mice per group) as follows: Group 1, chow diet (normal diet containing 4.8% fat (w/w), 0.65% Ca, and 0.5% P); Group 2, HFD (diet containing 45% fat (Jung Ang Lab Animal Inc., Seoul, Korea); Group 3, HFD + positive control (*Garcinia cambogia* (Gar) extract (165 mg/kg) with HFD); Group 4, HFD + positive control (orlistat (10 mg/kg) with HFD); Group 5, HFD + ST extract (100 mg/kg); Group 6, HFD + ST extract (300 mg/kg). Each morning, ST extract and positive controls were administered orally for seven weeks. During the study duration, the body weights and survival rates of the mice were examined daily. After seven weeks, the mice were anesthetized, and blood samples were collected to determine the following biochemical parameters: total cholesterol, insulin, triglyceride, and leptin levels. A portion of the hepatic and white adipose tissues (WAT) were immediately fixed in 10% formalin (Junsei, Tokyo, Japan), and then stained with hematoxylin and eosin (H&E) stain (Muto Pure Chemicals Co, Tyokyo, Japan). The remaining hepatic tissues and WAT were frozen in liquid nitrogen and stored at −70 °C for biochemical assays. All experiments were performed in accordance with the experimental animal guidelines of Jeju National University Animal Center (Jeju, Korea).

### 2.3. Serum Analysis

Blood samples were collected from the mouse heart by cardiac puncture into an EDTA-rinsed syringe. The blood was centrifuged to collect serum (12,902× *g*, 20 min, 4 °C). Commercial analysis kits were used to evaluate the content of serum triglyceride (TG), total cholesterol (TC), leptin, and insulin (Abcam, Cambridge, UK).

### 2.4. Hematoxylin and Eosin Staining

Paraffin block slides of pancreas tissues were deparaffinized, stained with Mayer’s hematoxylin solution (DAKO, Thetford, UK) for 30 s and eosin Y solution (Sigma-Aldrich, Saint Louis, MO, USA) for 10 s, rinsed three times with distilled water, and mounted using xylene-based DPX mounting solution. H&E stained tissue slide images were observed via light microscopy and the size of adipose tissues was calculated using Image J software (NIH, Bethseda, MD, USA).

### 2.5. RNA Extraction and Quantitative Real-Time Polymerase Chain Reaction

Adipogenic and thermogenic gene expression was analyzed via reverse transcription polymerase chain reaction (RT-PCR) techniques. mRNA was extracted from 30–40 mg mouse tissues using 1 mL Trizol reagent (Invitrogen, Carlsbad, CA, USA) placed in tubes containing 1mm of steel beads (Taco^TM^, Taichung, Taiwan). Tissues were then homogenized using a bead beater (tacoTM Prep bead beater) for five cycles (40 s/cycle). The homogenized mixtures were then centrifuged (25,482× *g*, 4 °C, 20 min). Supernatants were collected and the mRNA was extracted from the supernatant using phase separation and RNA precipitation. The isolated mRNA was reverse transcribed to complementary DNA (cDNA) using a cDNA reverse transcription kit (Takara, Shiga, Japan). Subsequently, the Thermal Cycler Dice Real-Time System (Takara, Shiga, Japan) was used to perform the reaction. cDNA amplification was conducted in optimal conditions (enzyme activation (95 °C for 10 s), followed by 40 cycles of denaturation (95 °C for 5 s) and extension (58 °C for 10 s)). The sequences for all primers were as follows: *GAPDH*: forward, 5′-TGTGTCCGTCGTGGATCTGA-3′, reverse, 5′-TTGCTGTTGAAGTCGCAGGAG-3′; *PPAR*: forward, 5′-GTCACGGAACACGTGCAGC-3′, reverse, 5′-ACTCAGAAGTGGGCGAGGAC-3′; *UCP1*: forward, 5′- CAGAGAGTTTGTCCTCTGGTGC-3′, reverse, 5′- GAAAAATCAAGGGTCCTGCCCC-3′. *UCP3*: forward, 5′- GTTTTGCTGATCTCCTCACCTTT-3′, reverse, 5′- GAACTGCTTGACAGAGTCATAGAGG-3′.

### 2.6. Statistical Analysis

All results were analyzed in triplicate and presented as the mean ± standard deviation (SD). Analysis of statistical significance was performed using statistical package for the social science software. Student’s *t*-test or one-way analysis of variance with Duncan’s multiple range tests were then performed. *p*-values < 0.05 were considered significant.

## 3. Results

Changes in body weight and fat accumulation in experimental mice.

**Figure 1:**
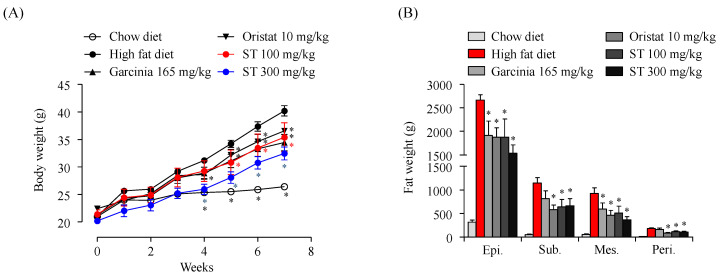
Effects of *Sargassum thunbergii* on body weight and fat accumulation. (**A**) Mouse body weight gain over seven weeks. (**B**) Weight of different types of fats. Epi: epididymal fat; Sub: subcutaneous fat; Mes: mesenteric fat; Peri: perirenal fat. All data is expressed as mean ± SD (*n* = 5/group). Significant differences were identified at * *p* < 0.05, as compared to the HFD group. ST: *Sargassum thunbergia* ethanol extract.

To evaluate the anti-obesity effects of ST, we measured the body weights of mice once per week throughout the study. Considering that *G. cambogia* extract and orlistat have reportedly beneficial anti-obesity effects [20,21], they were used as positive controls in the current study. Results show that the body weight of HFD group was significantly higher compared to that of the chow diet group (Figure 1A). Meanwhile, treatment with the positive controls inhibited the increase in body weight of HFD-induced obese mice. Supplementing the HFD diet with ST also resulted in significant reduced body weight compared to the HFD group. 

Further, we previously reported that indole derivatives isolated from ST inhibit adipogenesis in adipocytes. Therefore, we also measured changes in fat accumulation in the WAT of HFD-induced obese mice following ST treatment. We found that oral administration of ST at 100 or 300 mg/kg decreased fat accumulation in WAT (including epididymal fat (Epi), subcutaneous fat (Sub), mesenteric fat (Mes), and perirenal fat (Peri)) compared to that in the HFD group (Figure 1B). These results indicate that ST treatment can efficiently reduce body weight by inhibiting fat accumulation in WAT.

### 3.1. Effect of Sargassum thunbergii on Serum Biochemical Parameters

Excessive fat accumulation increases the risk of developing type 2 diabetes and cardiovascular disease in humans. Moreover, increased serum leptin levels are dependent on body fat accumulation. Meanwhile, HFD-induced obesity is characterized by an increased risk of type 2 diabetes with increased serum insulin levels. Obesity is a risk factor for cardiovascular diseases, and is associated with high levels of triglyceride and cholesterol in the serum [22]. To determine the anti-obesity effects of ST on biochemical parameters, we assessed the levels of serum insulin, triglyceride, TC, and leptin diabetes in Table 1. The level of insulin was found to be significantly higher in the HFD group compared to the chow diet group. However, it was significantly lower in the ST (100 and 300 mg/kg) + HFD groups compared to the HFD group. Additionally, significantly increased serum levels of triglycerides and cholesterol were detected in the HFD group compared to the chow diet group. Meanwhile the positive control (extract of *G. cambogia*) group reduced the triglyceride and TC levels in the serum compared to the HFD group. Similarly, treatment with ST (100 and 300 mg/kg) significantly decreased serum triglyceride and cholesterol levels compared with the HFD group. Furthermore, leptin levels were also markedly higher in the HFD group than in the chow diet group; while treatment with ST significantly decreased the serum level of leptin compared with the HFD group. These results indicate that ST treatment may be capable of improving metabolic disorders, such as cardiovascular disease and type 2.

### 3.2. Histological Analysis of Hepatic and Adipose Tissues

**Figure 2:**
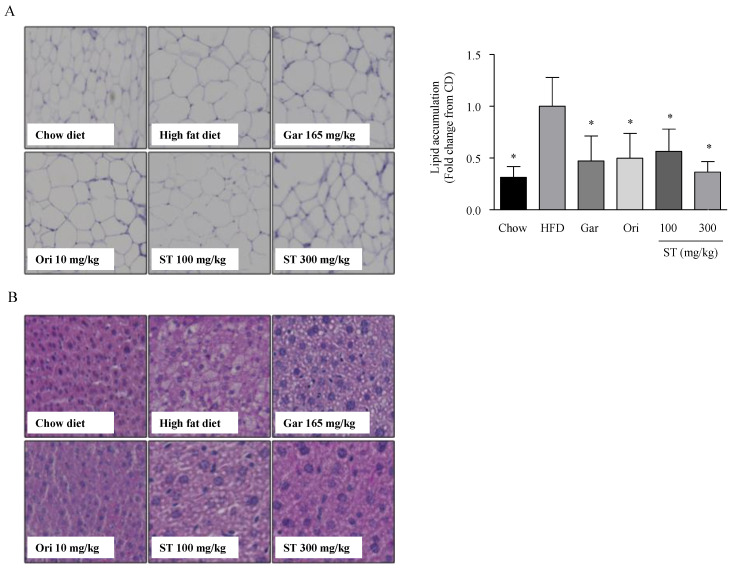
Histopathologic analysis of white adipose and hepatic tissues. (**A**) Histological changes in the central vein and portal area of white adipose tissue as assessed using Image J software. (**B**) Histological changes in the liver. The liver and white adipose tissue sections were stained with hematoxylin and eosin (H&E). Significant differences were identified at * *p* < 0.05, as compared to the high-fat diet (HFD) group. Gar: *Garcinia cambogia*; Gpo: *Garcinia cambogia* positive control; Ori: Orlistat; ST: *Sargassum thunbergii* ethanol extract.

Park et al. reported that HFD markedly increases the size and accumulation of fat tissue in fatty liver. Moreover, obese and overweight individuals are at an increased risk of developing fatty liver with higher amounts of WAT [23]. Fat accumulation in hepatic tissue and WAT was investigated using H&E staining. White adipocytes were observed to be larger in the HFD group compared to the chow diet group (Figure 2A). Meanwhile they were smaller in the HFD + ST (100 and 300 mg/kg) groups compared to the HFD group. Furthermore, a significant increase in the number of lipid droplets was observed in the hepatic tissues of the HFD diet group compared to the chow diet group. However, fewer lipid droplets were found in the HFD + ST (100 and 300 mg/kg) diet groups in a dose-dependent manner, when compared to the HFD group.

### 3.3. Expression of Adipogenesis- and Thermogenesis-Related Genes

**Figure 3:**
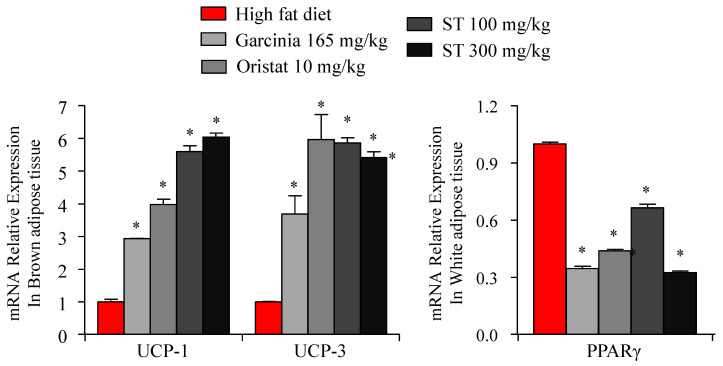
Effects of *Sargassum thunbergii* on the expression of adipogenesis (PPARγ) and thermogenic uncoupling protein 1 and uncoupling protein 3 (UCP-1, UCP-3) genes in white adipose tissue and brown adipose tissue. All data was obtained via RT-PCR analysis. Significant differences were identified at * *p* < 0.05, as compared to the HFD group.

The mitochondrial uncoupling protein 1 (UCP-1), and uncoupling protein 3 (UCP-3) play important roles in regulating energy expenditure. Specifically, activation of UCP1 and UCP3 leads to regulation of thermogenesis in brown adipose tissues (BAT) and inhibits fat accumulation by increasing energy consumption. Hence, we assessed the effect of ST on adipogenesis- and thermogenesis-related gene expression. Specifically, the mRNA expression of *Ucp1*, *Ucp3,* and peroxisome proliferator-activated receptor gamma (*Pparg*) was assessed via RT-PCR. BAT plays an important role in the regulation of energy balance by thermogenesis-specific genes, including *UCP-1* and *UCP-3* [24,25]. Results show that the mRNA expression of *Ucp1* and *Ucp3* in BAT was significantly increased in the HFD + ST (100 and 300 mg/kg) groups compared to the HFD group (Figure 3). In addition, treatment with positive controls (*G. cambogia* extract and orlistat) increased *Ucp1* and *Ucp3* mRNA expression in BAT compared to the HFD group. These results suggest that ST significantly increased energy consumption via thermogenesis during energy homeostasis in BAT. In addition, *Pparg* mRNA expression (an adipogenesis gene) was significantly decreased in the WAT of the HFD + ST (100 and 300 mg/kg) treated groups compared to the HFD group. These results further suggest that ST may reduce the risk of obesity by upregulating the expression of *Ucp1* and *Ucp3* in BAT while downregulating *Pparg* in WAT.

## 4. Discussion

Obesity is characterized by increased fat accumulation throughout the body and can lead to various complications, including increased risk of type 2 diabetes and hyperglycemia. HFD can cause metabolic syndrome, including obesity, type 2 diabetes, and hypertension [1,26,27,28]. In this study, we investigated the effects of ST on HFD-induced fat accumulation in the WAT of mice. Our results indicated that oral administration of ST decreased fat accumulation and body weight in HFD-induced obese mice. These results validate the results from a previous study in which indole derivatives isolated from ST inhibited adipogenesis through downregulation of adipogenesis-specific proteins in white adipocytes [19]. 

Obesity is also known to induce high levels of hematological parameters related to cardiovascular diseases, such as triglycerides and cholesterols in human serum [29,30]. Oral administration of ST caused a significant reduction in the serum levels of triglycerides and cholesterol when compared with the HFD group. These results indicate that treatment with ST had a beneficial effect on cardiovascular diseases in HFD-induced obese mice. Moreover, several studies have reported that obesity and overweight are the major risk factors for type 2 diabetes [31,32]. Our findings suggest that ST treatment may effectively reduce the risk of type 2 diabetes by controlling blood glucose levels in HFD-induced obese mice. Moreover, excessive fat accumulation is a marker of obesity, which can increase fatty liver and the size of white adipocytes [33,34]. Our results show that oral administration of ST in HFD-induced obese mice improved fatty liver and decreased fat accumulation. Adipose tissues play an important role in the regulation of energy balance and metabolic homeostasis. Various studies have shown that upregulation of the UCP gene in BAT inhibits fat accumulation and enhances energy expenditure. In particular, the expression of UCP1 is highly related to thermogenesis, which increases energy expenditure in BAT [35,36,37]. A number of studies have shown that extracts and compounds from natural substances inhibit fat accumulation through upregulation of UCP1 in BAT [38,39,40,41]. 

## 5. Conclusions

Our results indicated that ST might reduce the risk of obesity by upregulating lipolysis genes in BAT and downregulating the expression of lipogenesis genes in WAT. Taken together, these results suggest that ST may exert an anti-obesity effect by inhibiting fat accumulation in HFD-induced obese mice, and that it may be effective in functional food products for the treatment or prevention of obesity.

## Figures and Tables

**Table 1:** Effects of *Sargassum thunbergii* on serum levels of insulin, triglyceride, total cholesterol, and leptin.

	Chow Diet	High-Fat Diet	Gpo (165 mg/kg)	Ori (10 mg/kg)	ST (100 mg/kg)	ST (300 mg/kg)
Insulin (ng/mL)	0.86 ± 0.04 *	2.21 ± 0.62	1.76 ± 0.71	1.05 ± 0.28 *	0.96 ± 0.21 *	1.13 ± 0.42 *
Triglyceride (nmol/μL)	24.37 ± 2.34 *	30.65 ± 2.19	21.55 ± 3.33 *	25.40 ± 3.63 *	22.5 ± 2.54 *	23.28 ± 1.87 *
Total cholesterol (μg/μL)	69.02 ± 2.96 *	84.77 ± 4.05	67.24 ± 7.3 *	79.13 ± 5.55	74.59 ± 5.65 *	73.76 ± 1.61 *
Leptin (pg/mL)	425.60 ± 0.01 *	3521.6 ± 0.09	2218 ± 0.01 *	3638 ± 0.09	2859.6 ± 1.35	2299.6 ± 0.3 *

Within each treatment group, the means without a common letter differ significantly (* *p* < 0.05). Gpo: *Garcinia cambogia* positive control; Ori: Orlistat; ST: *Sargassum thunbergii* ethanol extract.
